# A model to facilitate critical thinking of radiography students

**DOI:** 10.1002/jmrs.697

**Published:** 2023-06-22

**Authors:** Tracey Pieterse, Annie Temane, Charlene Downing

**Affiliations:** ^1^ Department of Anatomy and Medical Imaging, School of Medical Sciences The University of Auckland Auckland New Zealand; ^2^ University of Johannesburg Johannesburg South Africa

**Keywords:** Critical thinking, problem‐solving, radiography education, reasoning, reflection

## Abstract

**Introduction:**

Critical thinking is a much‐needed skill required by radiography students, across disciplines, when they graduate. The facilitation of critical thinking is a task that radiography educators are faced with in order to produce graduates who can apply these skills in the clinical setting, for the best care of the patient. The development of critical thinking skills is challenging, and currently there is no radiography‐specific model which has been implemented and evaluated as a framework of reference for radiography educators. The aim of this article is to present a critical thinking model as a framework of reference that was implemented and evaluated by diagnostic radiography educators.

**Methods:**

A theory‐generating qualitative, exploratory, descriptive and contextual design was used for the development of a model to facilitate critical thinking skills of diagnostic radiography students in a South African setting.

**Results:**

A theory‐generation model to facilitate critical thinking skills for radiography students was developed. The model was implemented and evaluated by radiography educators. Three themes emerged from the evaluation of the model after implementation. The results indicated the implementation of the model provided a platform for radiography educators to collaborate and purposefully tailor activities to incorporate critical thinking into their teaching.

**Conclusions:**

Through the implementation of the model, radiography educators felt empowered by using a framework of reference to facilitate critical thinking skills of radiography students.

## Graphical abstract

Critical thinking is a much‐needed skill required by radiography students when they graduate. A theory‐generating qualitative, exploratory, descriptive and contextual research design was used for the development, implementation and evaluation of a model to facilitate critical thinking skills of diagnostic radiography students.

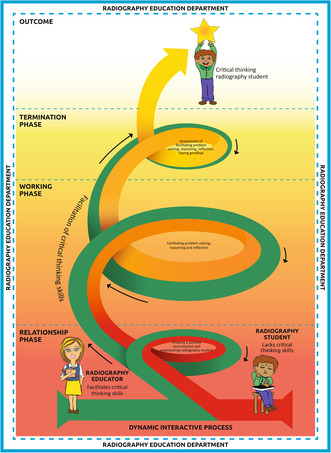

## Introduction

Internationally across health care, several studies report on the need to produce graduates with the ability to think critically.^1^, ^2^ Radiographers are required to actively use critical thinking skills to make sound clinical judgements, and the radiography curriculum falls short if it is not designed in such a way to instil these skills in the undergraduate years.^2^ However, literature relating to critical thinking skills within radiography has indicated diagnostic radiography students' ability to think critically is below the desired level.^3^, ^4^ In addition, barriers to critical thinking persist throughout radiography student's clinical placement as students are trained in a protocol‐driven environment.^5^, ^6^ This results in poor critical thinking and clinical reasoning in the clinical environment and ultimately has a negative impact on the health outcomes of the patient^3^ due to a reduction in the application of knowledge and limited clinical efficiency.^7^

Educators are responsible for forming critical thinking skills of students during their student years.^8^ This can be done through designing the curriculum by structuring teaching to include a wide variety of experiences that challenge learned knowledge and by creating tasks that are specifically aimed at developing critical thinking skills.^9^ Therefore, in addition to knowledge, educators need to instil critical thinking skills to assist students to navigate a variety of clinical situations effectively.^10^ Through this, students will be able to display critical thinking skills in the clinical environment for accurate clinical reasoning and to work effectively as part of the healthcare team for the best care of the patient.^11^

Critical thinking is a challenging skill to master,^11^, ^12^ and there is no clarity on how educators teach it within the healthcare profession.^13^ In addition, research on critical thinking in radiography is limited,^3^, ^14^ despite the vital need for these skills in the profession.^2^ Although a number of critical thinking models exist in health care, there is a need for the implementation of a radiography‐specific model to facilitate critical thinking as the definition of critical thinking differs for each profession.^8^, ^15^ Radiography educators need to be empowered to incorporate critical thinking skills within the radiography curriculum. This will meet the needs of professional boards who require graduates to demonstrate critical thinking skills^2^ as well as the healthcare team who relies on sound clinical decision‐making for the best care and outcome of the patient.

### Problem statement

Radiography educators are tasked with the challenge of incorporating critical thinking into their teaching to transfer these much‐needed skills to radiography students. Currently, there is no specific model that has been implemented and evaluated as a framework of reference to facilitate critical thinking skills in diagnostic radiography education.

### Aim

The aim of this article is to present a model that was developed and implemented as a framework of reference for diagnostic radiography educators to facilitate critical thinking skills of diagnostic radiography students.

## Methods

### Study design

A theory‐generating qualitative, exploratory, descriptive and contextual design was used in the study.^19^, ^20^ A model is a symbolic/diagrammatic representation of a theoretical relationship, achieved through words, pictures, diagrams, mathematical notation or physical structures.^21^ The implementation of the model was evaluated using a phenomenological approach through a focus group discussion in May 2021. Thematic analysis of data took place through Tesch's descriptive method of open coding as described by Creswell & Creswell.^22^

### Ethics

The ethical guidelines and research process were approved by the University of Johannesburg Research Ethics Committee and Higher Degree Committee (REC‐01‐116‐2016). The four pillars of bioethics, namely respect for autonomy, non‐maleficence, beneficence and justice^16^ were adhered to throughout the research process.

### Trustworthiness

Lincoln and Guba's model of trustworthiness was used to ensure credibility, transferability, dependability, confirmability and authenticity, respectively.^17^, ^18^ Credibility was ensured through prolonged engagement, persistent observation, triangulation and member checking. Dependability was ensured by providing a description of the research methodology, the code–recode method of data analysis, the use of an independent coder and an audit trail of the research process. Confirmability was achieved by ensuring reflexivity, using verbatim quotes, and an audit trail and authenticity was ensured through fairness by prolonged engagement, informed consent, member checking, reflexivity and the use of an independent coder.

### The development of the model

**Figure 1:**
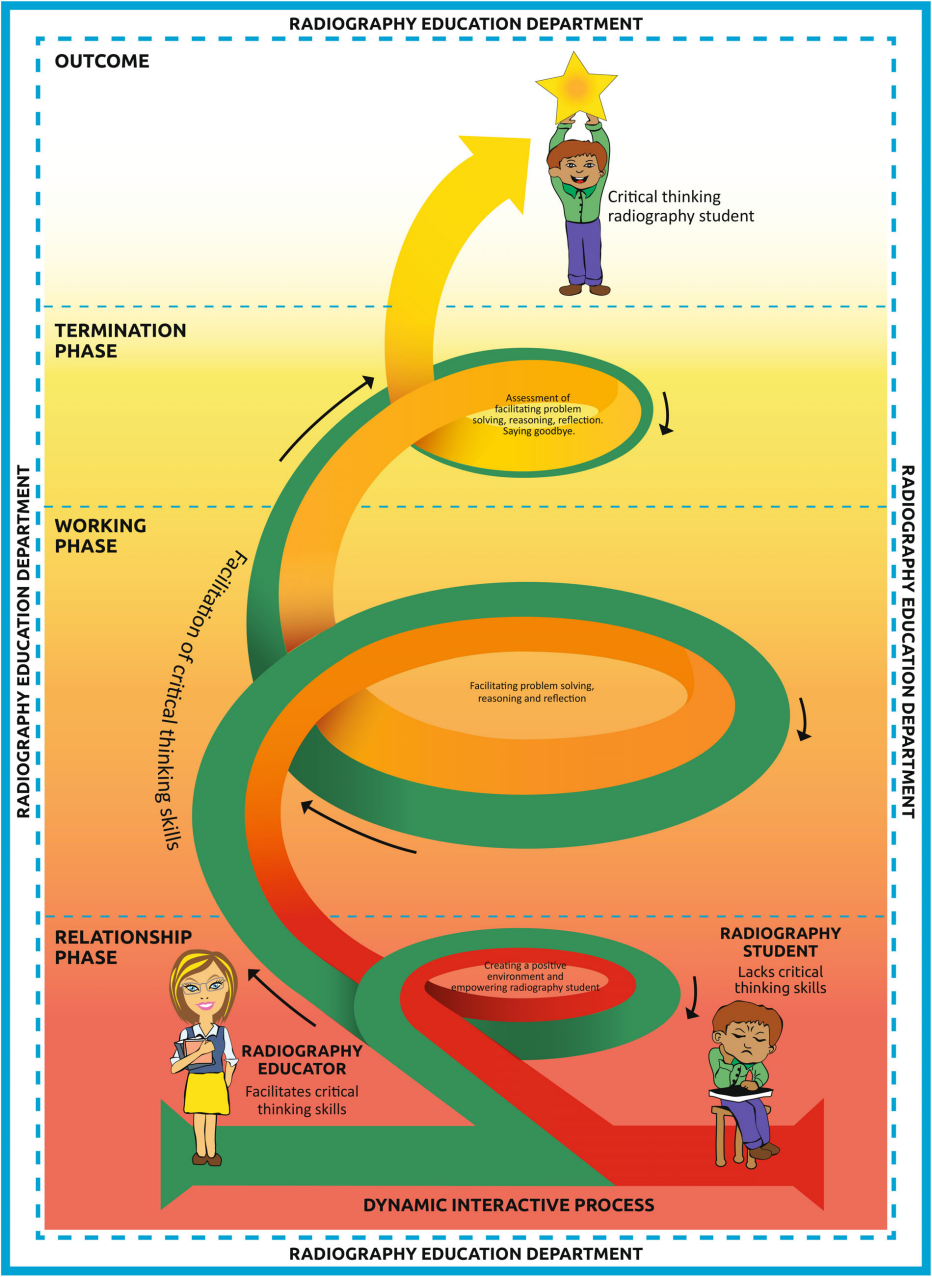
Model to facilitate critical thinking skills of radiography students.

The model (Fig. 1) was developed using the four steps identified from Chinn and Kramer's theory‐generation method.^20^ The steps include a concept analysis, relationship statements, a description of the model and an evaluation of the model by a panel of experts.^20^ By focusing on the identification and interpretation of critical thinking concepts within radiography, relationship statements were constructed, and a conceptual framework was created to assist in the development of the model for the facilitation of radiography students' critical thinking skills. For this study, critical thinking was defined as ‘the radiography student's ability to analyse, evaluate and problem‐solve in a clinical scenario in order to make a judgement based on evidence for the best outcome of the patient’.

### A model to facilitate critical thinking skills of radiography students

#### The relationship phase

The relationship phase is critical as the radiography educator and radiography student's relationship begins with a dynamic interactive process of engaging through the creation of a positive environment. The relationship phase of the model forms the foundation through the mobilisation of resources, mutual respect and trust and gives the radiography student confidence by empowering them. In the relationship phase the radiography student is provided with a safe learning environment that encourages active participation in classroom activities which are goal oriented through collaborative discussions, questioning and feedback.

#### The working phase

During the working phase, the radiography educator purposefully sets out a variety of learning activities to facilitate problem‐solving, reasoning and reflective skills of radiography students (Table 1), as they are exposed to learning new knowledge and link this to existing knowledge. Most of the facilitation is done during the working phase. Engagement occurs through a variety of teaching and learning activities purposefully set out by the radiography educator. These activities are carefully designed to promote problem‐solving, reasoning and reflection of radiography students. The activities will assist radiography students to think logically and make a judgement by analysing and evaluating evidence within context.

**Table 1:** Activities used during the model implementation for the facilitation of problem‐solving, reasoning and reflection.

1. Facilitating problem‐solving through problem‐based learning.	Activities were designed around common experiences students encounter in the clinical environment and based on the knowledge they have already gained during theory‐based classes. Problem‐based learning was used by giving students scenario‐based questions which led to a discussion.
1a) Simulation	Case‐based scenarios were used in a simulated setting. Simulation can include the use of mannikins, computer‐aided simulation software and simulated patients.
1b) Using triggers	Micro‐content such as text, images, illustrations, weblinks and games were used as triggers for a problem‐based learning activity. These triggers could be posted on learning platforms online or simply by using applications such as WhatsApp.
1c) Case studies	Carefully developed case studies were given to groups of students to work through. X‐ray images were included for students to analyse and evaluate within the context of the given clinical scenario.
1d) Example‐problem‐based‐learning (EPBL)^23^ and Plan‐Do‐Check‐Act (PDCA).^24^	Students in the lower levels of learning (first and second year) benefitted from EPBL. In EPBL students received examples of a problem‐solving activity which included three aspects^23^: The problem statement A variety of solution steps The final solution In the higher levels of learning (third and fourth year) students were encouraged to use the PDCA method of problem‐solving as a guide. The PDCA method of problem‐solving is an approach to identify the problem (P), establish a strategy (D), how to carry out the strategy (C) and then reflect on the results (A).^24^
2. Facilitating reasoning	Facilitating reasoning requires a combination of cognitive processes through gathering information and analysing a problem within its context, and then deciding on the actions that need to be taken. Reasoning only occurs at higher levels of thinking^25^, ^26^
2a) Metacognition	Educators were required to verbalise their thinking (metacognition). Probing questions were used to elaborate or explain in‐depth thinking processes. The ‘Plus, Minus and Interesting’ (PMI) approach to problem‐solving encouraged evaluative thinking and reasoning^26^ by considering: The positives (P) The negatives (M) Interesting questions or observations (I)
2b) Inquiry‐based learning (IBL)	Structured investigations were provided for students to ask and answer questions during a facilitator‐led discussion. Pictures, videos or texts were used as triggers to prompt discussions and questions
3. Facilitating reflection	Existing reflective models were used as a guide^27^ which began with a shared starting point that triggered an opportunity for reflection and included the following steps as a guideline: The situation: What, where and who? How did the situation make you feel? Why did the situation occur? What could be done differently? What will you do differently in future? Reinforcement by checking reflections against expectations and refining if needed.

In the model (Fig. 1), as the radiography student progresses, guidance from the radiography educator is needed much less as the radiography student becomes more independent and self‐directed in the termination phase. The radiography student has developed the skills needed for problem‐solving, reasoning and reflection in the final phase, and this leads to the outcome of the model where the radiography student becomes a critical thinking radiography student before they graduate.

## Results and Discussion

Due to COVID‐19 lockdowns, the activities in the working phase were adapted to be delivered in an online format using online teaching and learning software platforms, including simulation software. Limited opportunities existed during clinical placement visits to offer students face‐to‐face learning activities. The model was implemented and evaluated by radiography educators at a comprehensive university over three phases. Phase one took place immediately after a workshop informing participants of how the model should be implemented. Phase two of the evaluation took place after 1 month of implementation of the model, and phase three after 3 months of implementation of the model.

Six participants took part in the evaluation of the model implementation. This took place as a focus group discussion that was conducted online over Zoom. Table 2 summarises the demographics of the participants who took part in the model's evaluation after a full 3 months of implementation.

**Table 2:** Focus group participants' demographics.

	Participants (P)					
	P1	P2	P3	P4	P5	P6
Gender	Female	Female	Female	Female	Female	Male
Age	40	58	44	41	31	38
Highest qualification	Master's Degree	Master's Degree	Master's Degree	Master's Degree	Master's Degree	BTech Degree
Radiography education experience	14 years	15 years	8 years	4 years	3 years	2 years

The question asked of participants was ‘*How did you experience the implementation of the model to assist you in facilitating critical thinking skills of radiography students?*’

The central storyline which emerged after implementing the model was that it promoted a greater consciousness about teaching methods being underpinned by an educational philosophy. Online teaching posed challenges, but also great opportunities to encourage critical thinking in a creative and interactive way. Using different teaching methods to promote critical thinking is a disciplined process that should be scaffolded from the first year of study to the fourth year in radiography education.

Three themes emerged from thematic coding:

Theme 1: The model's implementation contributed to a greater level of consciousness about teaching methods already being underpinned by an educational philosophy
Sub‐theme A: When the model was implemented, participants became more conscious and reflective of their own teaching methods and engaged in peer collaboration

Participants found they were collaborating about their teaching approaches by discussing their various teaching approaches, creating teaching activities together and sharing their experiences with each other during team meetings and casual conversations. Participants also said they thought about their teaching approaches more carefully and purposefully, recognising what worked and what did not work well. Participants felt empowered when they realised the teaching methods, they were already using had a pedagogical underpinning. Pedagogical awareness enlightens educators about their teaching methods. This awareness allowed participants to evaluate their teaching approaches and refine their teaching methods.

With reflection, I did realise it's a lot of things we do already … but we don't necessarily know, we don't have that education background. (P3)

We actually do all these things, it's just that we didn't possibly have the right words or know that we were already implementing. (P5)

It is common for radiography educators with little to no educational background to take on academic teaching activities directly from a clinical environment. They rely on their professional experience and thus implement what is known as a “signature pedagogy”.^28^ Säde‐Pirkko and Asko^29^ confirmed that educators develop their teaching identity over time by constantly evaluating their teaching methods.

Participants felt they were consciously thinking about their teaching style. They considered how to purposefully change their teaching style and utilise the model to facilitate critical thinking.

I do this, but then I, after, after our session I'm like, how can they do this right. (P1)

I give them topics … and they [students] had to think about how to bring across the justification of why you would do this modality over that one and all those kind of things. (P2)

The model provided a platform to create a positive environment and improve relationships with radiography students. Open conversations enabled radiography students to feel comfortable enough to share thoughts and ideas during classroom discussions.

I really was interested to know how they [students] were … the ones that told me how they were, now I noticed on the online platform. They are the ones that put up their hands or say something, because they feel they can talk to me. (P1)

I think something that um, made the relationship better was ‐ when you share things, mistakes you've made or things that you've done, then all of a sudden they're like ‘oh right, right’ … ‘oh they're humans’ and we're [radiography educators] also radiographers … that sharing of experiences makes it a stronger relationship. (P2)

Educators contributing their own personal stories and clinical experiences make the classroom authentic as part of a sharing culture to foster engagement, mutual respect and trust.^30^ Collaborative conversations enable students to feel comfortable enough to share thoughts and ideas.^5^ Students who have a positive relationship with the educator are more motivated and therefore have higher educational aspirations and better academic success through intellectual development.^31^

Sub‐theme B: Adjustments to teaching and learning methods encouraged critical thinking and problem‐solving competency

Participants found themselves purposefully designing activities with careful thought. Participants used questioning as a way to encourage radiography students to think critically and justify their thinking.

I phrase questions differently – just changing the questions alone, I think it makes them think about different aspects. (P3)

They [students] would ask a question … and we would respond with a question … but then after a while, I think they [students] enjoyed us asking them question upon a question, because then the the one even said “oh thank you for telling me I went and read that up”(P1)

Quality thinking is encouraged when students become self‐directed to find their own answers to questions, guided by the facilitator.^32^ This teaching method helps students become creators of knowledge rather than consumers of knowledge, promoting their problem‐solving and critical thinking skills.^33^

Participants used the model as a guide to give radiography students more responsibility in their learning, by assigning them teaching and learning activities. A variety of tasks were used to improve radiography students' engagement during teaching sessions.

I assign the students that are going to be giving tuts [tutorials] to us. (P6)

I tried role‐play … I have been trying flipped class … [when] it's one of them [students] presenting then they tend to be able to ask more questions. They tend to not be afraid to try and respond when the student is asking questions. (P4)

According to Theobald et al.,^30^ a knock‐on effect of student engagement is an improvement in graduate outcomes by encouraging class attendance and participation. Students find collaborative engagements conducive to learning and help increase their understanding, encouraging inclusiveness in the cohort.^34^

Participants used questioning techniques to motivate radiography students to think about their answers and the justification behind their answers. This led to radiography students taking more responsibility for their learning and becoming reflective and independent thinkers.

I always ask them “so what does it mean? So you've written it, but what does it mean?” For example, like central ray or anode heel effect, but what does it mean to you … practically what does it mean to you … (P5)

Questioning techniques are associated with increased student motivation as students take charge of their learning by taking responsibility and becoming authors of their answers.^35^ This approach to teaching assists students in developing their own understanding through knowledge discovery and interaction, which fosters independent learning.^28^

Theme 2: Online teaching posed challenges, but also great opportunities to encourage critical thinking in a creative and interactive way

Multiple challenges were faced in teaching online during the COVID‐19 pandemic, but participants used them to develop new teaching methods, embracing the available opportunities.

Sub‐theme A: Participants faced challenges with online teaching

Participants felt detached from their students during online teaching and learning. Radiography students seemed to be less motivated to participate during online sessions as opposed to face‐to‐face classes.

You know, you have a class of 90 students, for example, and only 40 students consistently attend the lectures. We don't know when the students are actually listening to the class, if they actually are, so you know that has been quite a challenge. (P3)

You know, you say, “so what do you see” … and you get nothing. (P2)

Cavinato et al.^34^ found that 33% of students are less willing to answer questions during a Zoom session, 30% are less willing to participate in class activities online, and 36% are less willing to ask questions as opposed to face‐to‐face lectures. Moving to online teaching caught participants off guard, and all software tools available to participants may not have been fully utilised. Mardiana^32^ identifies this as a problem caused by the rapid move to online teaching, meaning educators did not have enough time to equip themselves with the knowledge of available online tools and how best to utilise these to increase classroom interaction.

Participants felt significant frustration and demotivation with online teaching due to the inability to see their radiography students face‐to‐face stating they felt alone in the teaching session.

Ya, because you are speaking to this black screen – and only five students. And you know, it's only those students all the time that will respond. (P4)

with the online, it is so much harder to do these kinds of things … when you can't see somebody. (P2)

Notably, not all students have the same accessibility or funds to fully facilitate the move to online learning.^36^ In addition to a lack of digital infrastructure, students are also faced with family responsibilities, health and mental wellness issues, as well as food and financial insecurities.^34^ These are some of the issues that may have plagued radiography students during online teaching sessions. It can be surmised that due to family demands or data restrictions, students may have no option but to turn off their video cameras to attend lectures. Cavinato et al.^34^ found that 39% of students cited some of these reasons affected their ability to fully participate in online classes.

Sub‐theme B: Different opportunities arose from online teaching

Although challenging, the move to online platforms meant participants became creative and innovative in their teaching approach, finding new ways to engage radiography students in their teaching sessions.

But what I did use in, in, during lectures, was the polls that you can do. So, then I'd ask a question, and have like a couple of responses. And then they [students] like doing that because that's anonymous … So polls are much better … You need to be innovative… I think innovation is key. (P2)

You have to come up with all these weird and wonderful things to get them [students] engaged. (P5)

Considering the known barriers students face related to online learning means educators can find strategies in their teaching to overcome these barriers.^34^, ^37^ Van Wyk et al.^38^ described this as ‘disruptive innovation’. Disruptive innovation was first described in education when the speed of technology increased rapidly, forcing educators to engage with and implement technology in their teaching approaches.^38^

Participants became creative in their delivery of content and questioning techniques to overcome the barrier of disengaged radiography students. Participants used technology to assist in the model's implementation by asking questions on WhatsApp and using tools in the online learning management system.

What I did was, I would put up activities of a WhatsApp group. And, and then they could respond. (P3)

it is quite inspirational to take something that is challenging … I think it can be quite inspiring to see when something works. You know, like now you've tried something and it's, it inspires you to do more. (P2)

Creating online resources to assist radiography students find information, adding frequently asked questions and posting reminders assists those who do not have a natural motivation but need guidance to become motivated as they succeed.^39^ Misra and Mazelfi^40^ found WhatsApp to be a beneficial form of communicating with students, since it is freely available and allows instant communication with multiple students at once. Although remote learning is a challenge for students with limited resources, many students have access to WhatsApp, which is easily integrated into teaching small topics or sharing images and videos.^38^

Participants said radiography students interacted with each other more when they prepared their own content to deliver to their peers.

As soon as I put another student to be the one teaching it sort of prompted them to speak more to differ with the student more. (P6)

they [students] lecture the first term … they've prepared all of the lectures … they did it incredibly well. (P2)

the confidence, it differs when it's someone else that's presenting other than you, the lecturer. (P4)

Giving students self‐directed tasks increases their autonomy and motivation to engage with the group.^41^ Mpalyani et al.^42^ acknowledge that self‐directed learning is known to motivate students, with the result of promoting lifelong learning. In line with this, Misra and Mazelfi^40^ agree that students who can work independently become motivated and cognitively strong.

Through the model's implementation, participants increasingly collaborated with each other to discuss ideas and teaching methods. Participants recognised this as a positive outcome of the model implementation process that was unexpected. Participants also felt the model allowed them to purposefully engage with radiography students through various teaching techniques.

They [students] think that whatever you are saying is always right, but I mean we [are] also in a learning process together with them [students]. (P4)

one more thing about this model [implementation] … that I think we would like, speak to each other more like “what are you going to do or what have you done?” (P1)

Vilppu et al.^28^ recognised collegial collaboration plays a vital role in education by creating a community for discussion around teaching techniques and enabling educators to feel less isolated in their pedagogical journey. Positive engagement with students is aligned with academic success through the creation of an environment of open communication.^38^

Theme 3: Applying different teaching methods to promote critical thinking is a developing disciplined process, which should start in the first year of study and continue through to the fourth year of study

Participants indicated that the model implementation process needs to be scaffolded from year one to year four and should be considered a long‐term process in order to gain maximum benefit.

Sub‐theme: Critical thinking development

Participants found the model's implementation gave them a foundation through which they could develop radiography students' critical thinking skills. They indicated the need to work together to strategically implement teaching and learning activities throughout the curriculum, specifically aimed at each year level to purposefully scaffold the teaching of critical thinking from the first year to the fourth year to maximise the benefit of the model's implementation.

But for the first years we really had to be like focus on getting the concepts right, the foundation right or else they're gonna struggle. (P5)

I haven't in first year given a full scenario. I'm going to do it now, because I thought – build it up slowly. (P3)

I feel like it's too soon to leave them … I feel like it's a process … so if we're starting now consciously, so next year the second years are going to be in a different phasẹ (P1)

When you move on first to fourth year, you have to consider the level of students, and how we implemented it. (P3)

According to Kirmizi et al.,^43^ critical thinking skills should be developed from the start of an education programme. In order to understand and base decisions on evidence, students should receive new information that can be connected to previous knowledge in line with constructivist learning approaches.^44^ Careful curriculum coordination is integral in developing critical thinking skills by scaffolding learning throughout the programme.^45^

Through the implementation of the model, participants guided radiography students towards self‐directed and independent learning.

I send them questions ahead of time, so they can prepare and come prepared for discussion … students have to then also engage in, in creative thought to think beyond what they normally know and [think] lateral. (P3)

I've asked them [students] to go away and reflect on kind of, their clinical practice related to what we've been doing. (P2)

Learner‐focused approaches to teaching are known to encourage students' critical thinking by discovering knowledge on their own.^28^ Self‐directed learners are more successful in achieving learning outcomes. With the introduction of online teaching during the COVID‐19 pandemic, self‐directed learning will become increasingly relevant.^46^

## Limitations

The impact of COVID‐19 meant that many of the face‐to‐face teaching strategies in the implementation of the model could not take place. Instead, face‐to‐face activities were re‐designed in an online format utilising the online learning platform. Additionally, the workshop to assist radiography educators to implement the model had to take place online rather than face to face.

## Conclusion

The development of critical thinking skills before radiography students graduate is necessary to ensure the best outcome for the patient in a radiography setting. Through a theory‐generation process, a model was developed for implementation to assist diagnostic radiography educators in the facilitation of critical thinking skills of radiography students.

## Conflict of Interest

The author declares no conflict of interest.

## Data Availability

The data that support the findings of this study are available from the corresponding author upon reasonable request.
